# Wannier Function Localization Using Bloch Intrinsic Atomic Orbitals

**DOI:** 10.1021/acs.jpca.4c04555

**Published:** 2024-09-19

**Authors:** Andrew Zhu, David P. Tew

**Affiliations:** Physical & Theoretical Chemistry Laboratory, University of Oxford, Oxford OX1 3QZ, U.K.

## Abstract

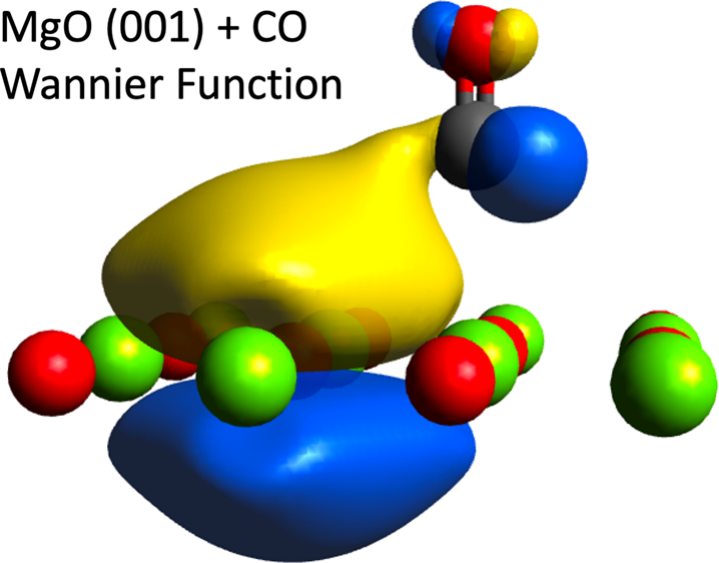

We extend the intrinsic atomic orbital (IAO) method for the localization of molecular orbitals to calculate well-localized generalized Wannier functions in crystals in the spirit of the Pipek–Mezey method. We furthermore present a one-shot diabatic Wannierization procedure that aligns the phases of the Bloch functions, providing immediate Wannier localization, which serves as an excellent initial guess for optimization. We test our Wannier localization implementation on a number of solid-state systems, highlighting the effectiveness of the diabatic preparation, especially for localizing core bands. Partial charges of Wannier functions generated using Bloch IAOs align well with chemical intuition, which we demonstrate through the example of the adsorption of CO on a MgO surface.

## 1 Introduction

Mean-field theories, such as the Hartree–Fock (HF) or Kohn–Sham density functional theory (DFT),^1,2^ provide a description of the electronic structure of a system through a one-particle orbital model, giving insights into the bonding in molecules and the band structure for materials. However, the canonical molecular orbitals (MOs) or Bloch functions are typically delocalized across the entire system and thus do not intuitively map to the interpretation of bonding in terms of overlap of atomic orbitals (AOs), which is a local picture. By applying unitary rotations to the occupied orbitals, one can obtain localized objects, commonly known as Wannier functions (WFs)^3^ for periodic systems. Localization of occupied orbitals aids in the interpretation of the electronic structure and also provides a basis for reduced scaling quantum chemistry methods, which exploit this locality to truncate the virtual space.^4−7^

Methods to evaluate localized MOs have focused on defining a localization metric or functional; the stationary points of this functional thus correspond to localized orbitals. The two most commonly employed metrics are from Foster and Boys (FB)^8,9^ and Pipek and Mezey (PM),^10^ both of which have been adapted for periodic systems.^11−16^ The FB metric, which minimizes the spread of the orbitals, has seen widespread usage, namely, through the Wannier90^12^ package, which has now established interfaces with various periodic, plane-wave-based codes.^17−20^ In contrast, the PM metric, which uses the Mulliken partial atomic charges, is naturally suited to codes employing localized basis sets, under a linear combination of atomic orbitals (LCAO) framework,^21−25^ where AO coefficients are directly accessible and overlaps are easily computed. In addition, WFs localized with the PM metric produce orbitals with separate σ and π bonding characters, giving advantages in chemical interpretation, as opposed to FB. The Mulliken charges, however, are unreliable for nonminimal basis sets. This issue arises from the near-redundancy of LCAO expansion with large basis sets and is exacerbated in crystals. To avoid this problem, alternate partial charge definitions^14,15^ have been utilized to obtain localized WFs in the spirit of the PM method. The intrinsic atomic orbital (IAO) method^26^ is one partial charge estimate that has successfully been applied to molecules.

In this paper, we introduce Bloch IAOs as the natural periodic extension of IAOs and then present the overall optimization scheme to generate localized WFs with Bloch IAOs. By generalizing the well-established IAO method to crystals, a direct comparison of localized orbitals between periodic and molecular systems becomes feasible. The initial guess is a crucial step in the optimization, and we propose a simple and effective procedure for generating localized orbitals by defining a natural gauge and by constructing diabatic Bloch orbitals and diabatic WFs. We then present and analyze the performance and stability of the optimization, with a particular discussion of the solver’s performance when separating core and valence bands. Finally, the chemical interpretability of Bloch IAOs is commented upon, using a surface adsorption system as an example.

## 2 Theory

### 2.1 Review of WFs

In the context of the LCAO framework, the crystal orbitals are expanded in the basis of Bloch AOs under Born–von Karman (BvK) boundary conditions. The Bloch AOs are eigenfunctions of the momentum operator with crystal momentum wave vector **k** and are defined as
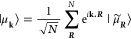
1where *N* is the number of unit cells within the BvK “supercell” and ***R*** is the lattice vector of the unit cell. |μ̃ _***R***_⟩ is the infinite sum of real-space AOs over the lattice vectors of the supercell and is defined as
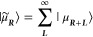
2

The crystal orbitals, also referred to as Bloch functions, are eigenstates of the one-particle Hamiltonian of a periodic system and are given by
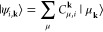
3The Bloch functions are delocalized across the entire system. By superimposing the Bloch functions of a single band across the first Brillouin zone, a conventional WF,^3^ centered on a unit cell given by lattice vector ***R***, is given by,
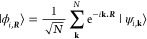
4The WFs span the same space as their Bloch counterparts, with translational copies found in each unit cell.

Bloch functions are defined for an arbitrary phase only. However, the spatial distribution of the resultant WFs is highly dependent on the relative phases of the contributing Bloch functions. The WFs are thus gauge variant. To obtain localized conventional WFs, the relative phases of the Bloch functions for each band must be optimized. By rotating the gauge such that the Bloch functions appear smooth in the reciprocal space, the resulting WFs in the real space are in turn localized, as a property of Fourier transforms,
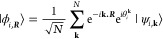
5A natural gauge for each Bloch function can be defined by requiring that the scalar product of the coefficients between Bloch functions at **k** and the Γ-point **0** is real-valued. By first computing the phase difference,
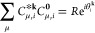
6the Bloch functions can be rotated into their natural gauge |ψ_*i*, **k**_^n^⟩, from their original gauge, |ψ_*i*, **k**_^o^⟩, straightforwardly,
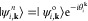
7For bands that have small dispersion, or minimal mixing, imposing the natural gauge is often sufficient to produce well-localized WFs.

### 2.2 Generalized Localized WFs

Generalized WFs are defined by allowing Bloch functions from several bands to mix,^11^
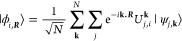
8By allowing mixing between bands, WFs can be further localized not only to a unit cell but also to atomic sites within a unit cell. The resulting generalized WFs are even more strongly “nonunique” than the conventional definition, and their locality is highly dependent on the choice of *U*_*ji*_^**k**^. To ensure real-valued WFs, an inversion symmetry about the Γ-point must be imposed between the Bloch functions |ψ_*i*,**k**_⟩ = (|ψ_*i*,–**k**_⟩)*. The Bloch functions at the Γ-point are real, following convention, and the choice of unitary is governed by the constraint of ***U***^**k**^ = (***U***^**–k**^)*.

Localization of WFs is achieved by varying ***U***^**k**^ to optimize a chosen locality metric. The FB^8,9^ and PM^10^ metrics are two of the most important examples. The FB method localizes orbitals by defining a metric that minimizes the orbital spread, as given by its variance,
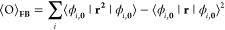
9Marzari and Vanderbilt^11^ generalized the FB approach, originally conceived for molecules, to evaluate localized WFs, creating the so-called FBWFs. The Wannier90 package,^12^ which employs this method, has been widely used among the solid-state community.^13^

The original PM metric was defined as the sum of squares of the Mulliken partial charges.^10^ To generate localized WFs using the PM metric (PMWFs), the objective functional is given by,
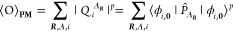
10where *Q*_*i*_^*A*_***R***_^ is the Mulliken charge^10^ associated with WF *i* on atom *A*, situated in unit cell ***R***, evaluated using the WFs located in the reference unit cell.  projects onto a basis of AOs centered on atom *A*_***R***_, given by,
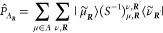
11where *S*_μ,***R***_^ν,***R′***^ = ⟨μ̃ _***R***_|ν̃ _***R′***_⟩ is the overlap. Under a BvK LCAO approach, where AO coefficients and periodic AO overlaps are naturally available, computing the PM metric is extremely straightforward as opposed to the FB metric, which has been used more commonly with plane-wave basis sets. In addition, the strong interpretability of PMWFs, producing orbitals with σ and π separation,^14^ as opposed to the “banana” bonds found within the FBWF scheme, further motivates our choice to utilize this metric.

The value of the penalty exponent, *p*, is typically 2 or 4. If *p* is chosen to equal 1, then eq 10 reduces to the normalization criteria of the bands,
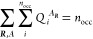
12where *n*_occ_ is the number of occupied bands.

A key issue with the original definition of the PM metric is that the Mulliken partial charges do not possess a complete basis set limit, meaning they are unreliable for nonminimal basis sets. Alternative charge definitions for MOs have been suggested,^27−29^ which remove this basis set dependence. Lehtola and Jónsson demonstrated that the localized orbitals obtained were largely independent of the chosen partial charge estimate,^27^ providing significant freedom in choice. In the context of periodic systems, Jónsson et al.^14^ first introduced a scheme to generate PMWFs, avoiding the issues surrounding Mulliken charges by using real-space partitioning of orbital charge densities. Clement et al. later outline an alternative charge definition based on projection onto a predetermined set of minimal basis functions.^15^

The IAO method, as proposed by Knizia,^26^ is one choice of an alternative partial charge estimate that has been employed successfully for molecules. Using a free-atom minimal basis as a template, contraction coefficients from the original basis to IAOs are defined such that the occupied orbitals are exactly represented, which provides a consistent assignment of the charge to atomic centers. Localized MOs using IAOs align well with chemical intuition, and quantitative measures such as partial charges and populations are shown to be resistant to changes in the original basis, and are consistent with chemical understanding, leading to the method being implemented in many quantum chemistry packages.^24,25,30,31^ We thus propose to adapt IAOs to construct a charge metric suitable to localize WFs, *Q*_*i*_^*A*_***R***_^IAO^^, using Bloch IAOs.

### 2.3 Bloch IAOs

Given the success of IAOs within molecular schemes, we believe that a **k** space extension to periodic systems would be desirable. Having the same partial charge estimate for both molecular and periodic systems opens the possibility of making direct comparisons across systems. Schäfer et al.^32^ demonstrate the use of IAOs to evaluate localized WFs for a Γ-point-only calculation, following the molecular formulation as described by Janowski.^33^ Cui et al.^34^ construct crystal IAOs, from which projected AOs are evaluated. We employ similar principles in our generalization to **k** space but crucially outline the additional augmentations needed to construct localized WFs, optimized using the PM metric, as a full periodic adaption of the IAO method.

We choose to adapt Knizia’s method,^26^ such that a set of IAOs are constructed for each **k** point within our Monkhorst–Pack quadrature mesh.^35^ The Bloch IAOs are able to exactly describe the original occupied Bloch functions, providing a basis independent charge metric for WFs.

The original Bloch functions (eq 3) are expressed in terms of Bloch AOs in the original basis set, labeled *B*_1_. Analogous to Knizia’s approach, a minimal basis, *B*_2_, of free-atom AOs is first chosen, from which corresponding Bloch AOs are obtained, |ρ_**k**_⟩ where ρ ∈ *B*_2_, from eq 1.

The following projection operators are defined,
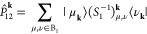
13
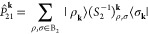
14where (*S*_1_)_μ,ν_^**k**^ = ⟨μ_**k**_|ν_**k**_⟩ and (*S*_2_)_ρ,σ_^**k**^ = ⟨ρ_**k**_|σ_**k**_⟩ are the Bloch AO overlap matrices in the original and minimal basis sets, respectively. Using these operators, depolarized occupied Bloch functions are obtained through
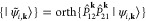
15or in the matrix form:
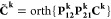
16Here, orth{} denotes symmetric orthogonalization and the transfer matrices are **P**_**12**_^**k**^ = (**S**_1_^–1^)^**k**^**S**_12_^**k**^ and **P**_**21**_^**k**^ = (**S**_2_^–1^)^**k**^**S**_21_^**k**^, where (*S*_12_)_μ ρ_^**k**^ = ⟨μ_**k**_|ρ_**k**_ ⟩ and **S**_21_^**k**^ = **S**_12_^**k**†^. The projector onto the depolarized occupied Bloch functions is Õ^**k**^ = ∑_*i*_|ψ̃ _*i*,**k**_⟩⟨ψ̃ _*i*,**k**_|.

The Bloch IAOs are the minimal Bloch AO basis that contains both the depolarized and polarization contributions and are defined through
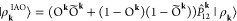
17In matrix notation, eq 17 is given as
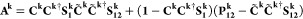
18where **A**^**k**^ is the contraction coefficient from Bloch AOs to Bloch IAOs at each **k** point and **1** is the identity in the space of *B*_1_. Janowski^33^ and Knizia^26^ both outline a simpler definition for the IAOs, which is equivalent under the assumption that *B*_2_ can be directly expressed in *B*_1_. Having implemented both schemes, we note that the output Bloch IAOs are very similar, with no significant difference in localization performance. Finally, the coefficients of the occupied Bloch functions in the Bloch IAO basis are given by
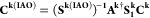
19
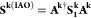
20In the original molecular implementation, the output IAO coefficients are symmetrically orthogonalized. However, in the periodic case, we chose not to do so. The orthogonalization procedure introduces arbitrary phases to the Bloch functions in the IAO basis, specifically when obtaining the eigenvectors of the IAO coefficient matrix. The relative phase differences between **k** points are thus altered compared to the original Bloch functions expressed in *B*_1_, leading to issues when optimizing the set of unitary matrices, across the Brillouin zone, in the IAO basis, since they do not correspond to the original Bloch functions. The simplest solution to remove this additional gauge problem is to leave the IAOs unorthogonalized. The “depolarized” Bloch functions, given by eqs 15 and 16, which are orthogonalized, avoid this issue because any phase augmentation is canceled in the projector Õ^k^.

In summary, obtaining the Bloch IAOs is numerically straightforward, requiring only a free-atom basis, and its corresponding Bloch AO overlaps to perform the matrix multiplication steps. Computation of inverse overlap matrices can be avoided by solving instead with the Cholesky decomposition. The Bloch coefficients in the IAO basis and the IAO overlap matrix can then be used in PM-style optimization to obtain optimally local WFs. The PM projector (eq 11), in the Bloch IAO basis, is now defined as
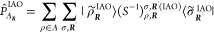
21where *S*_ρ,***R***_^σ,***R′***(IAO)^ = ⟨ρ̃ _***R***_^IAO^|σ̃ _***R′***_^IAO^⟩ is the IAO overlap in the real space, obtained from Fourier transforming **S**^**k(IAO)**^.

It should be noted that Clement et al.^15^ also employed a minimal basis of free-atom AOs to calculate a robust charge estimate. Orbital coefficients were obtained by computing the pseudoinverse of the overlaps between the Bloch functions in the original basis and the real-space reference cell AOs in a minimal basis. While this charge estimate is also simple to evaluate and demonstrated to be robust and basis set-resistant, Bloch IAOs have the additional advantage of being able to exactly represent the occupied space, as demonstrated first in molecules.

### 2.4 Localization Procedure

Recent work obtaining PM localized WFs and MOs has involved optimization algorithms to determine the stationary points of the functional.^14−16,27,36^ Schreder and Luber^16^ implemented a method that simultaneously applies complex Jacobi rotations to several unitary matrices^37^ in order to maximize the PM functional. Clement et al.^15^ recently demonstrated that a solver using the Broyden–Fletcher–Goldfarb–Shanno (BFGS) algorithm leads to significantly faster convergence compared to the previous steepest ascent (SA) or conjugate gradient implementation. Our localization procedure uses a BFGS-based algorithm that we employ in conjunction with our Bloch IAO charges. To generate an effective initial guess for the optimization, a novel procedure generates approximately localized WFs, which we call diabatic WFs.

#### 2.4.1 Diabatic Wannierization

The initial guess for the WFs is an important step in the localization procedure in order to avoid encountering local maxima. Methods that project Bloch functions onto a set of trial functions have been outlined,^11,13^ while other implementations ensure that the unitary space is probed fully by running multiple calculations using randomly sampled unitary matrices.^14,15^ Clement et al.^15^ combine random unitary sampling with a procedure to remove the gauge freedom of the Bloch functions.

As mentioned previously, Bloch functions are defined with an arbitrary gauge (eq 5). By fixing the gauge such that the variations between Bloch functions in **k** space are gradual, the Fourier transform produces WFs that are largely localized to a single cell, serving as an excellent starting guess for further optimization. We defined the natural gauge to be where the scalar product of the coefficients between Bloch functions within a band at **k** and the Γ-point **0** is real. For generalized WFs (eq 8), where the gauge uncertainty is increased by mixing bands, we extend the intuition of the natural gauge to construct diabatic Bloch orbitals and diabatic WFs. First, the Bloch orbitals of the Γ-point are localized by orthogonal transformation,
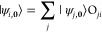
22The Bloch orbitals of the remaining **k** points are then chosen to be those with maximal similarity with the Γ-point. The locality of the orbitals of the Γ-point is thus transferred diabatically across the first Brillouin zone. This is obtained by calculating the unitary matrices, outside the Γ-point, which give the minimal least-squares difference to the Bloch coefficients of the Γ-point,
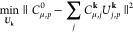
23where ||···|| is the Frobenius^38^ norm. As this is an example of an orthogonal Procrustes problem,^39^ a solution can be easily obtained via the singular value decomposition of the product of Bloch coefficients ***C***^***k*†**^***C***^**0**^,
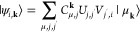
24
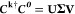
25In this work, we employ a convenient approximate localization procedure for the Bloch orbitals of the Γ-point, where we simply replace them with the Cholesky vectors of the Γ-point density, ensuring that computation of the diabatic WFs is fast.

#### 2.4.2 Optimization of the PM Metric

We implement a gradient-based optimization method to obtain the stationary points of the PM functional. Similar to the study of Clement et al.^15^ and Lehtola and Jónsson,^36^ a Riemannian geometry approach is adopted to maintain the unitary constraint, as outlined in refs (40,41). This method has proved successful since the unitary constraint is maintained implicitly, while other methods, including Lagrange multipliers,^42^ may suffer from slow convergence or only obtain a solution that only approximately maintains orthonormality.

Given the extensive discussion of the unitary optimization algorithm in refs (40,41), we only briefly outline our procedure here. Crucially, the PM charge metric and all associated expressions are evaluated in the Bloch IAO basis, using **C**^**k(IAO)**^ (eq 19). The real-space IAO overlaps, *S*_σ, ***R′***_^ρ, ***R***(IAO)^, are also used.

In the following expressions, the IAO labels are omitted for the sake of clarity. The Bloch IAO charges are defined as
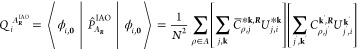
26where we have introduced *C*_ρ,*j*_^**k**,***R***^ = *C*_ρ, *j*_^**k**^ e^*i***k**·***R***^ and *C̅*_ρ,*j*_^**k**, ***R***^ = ∑_σ,***R′***_*C*_σ,*j*_^**k**,***R′***^*S*_σ,***R′***_^ρ,***R***^. The Euclidean derivative of the PM functional, ⟨O⟩_**PM**_, with respect to the unitary at **k**, is given by
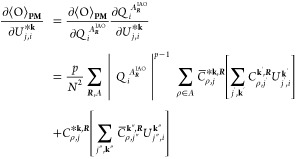
27

The Riemannian gradient, ***G***_**k**_, can then be transformed from the Euclidean gradient, , by
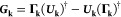
28The “two-loop recursion” version of the limited-memory BFGS algorithm (l-BFGS)^43^ is used, as first implemented for WFs by Clement et al.,^15^ to obtain a search direction, {***H***_**k**_}. The matrix elements in the upper triangle of the anti-Hermitian matrices for the Riemannian gradient, {***G***_**k**_}, form the gradient vector for the l-BFGS algorithm, ensuring that the output search direction is located on the unitary manifold. Given the requirement for the output WFs to be real, as mentioned earlier, our gradient vector is composed of the total *o*^2^(*N* – 1)/2 + *o*(*o* – 1)/2 real numbers, where *o* is the number of Bloch functions being localized.

To obtain a suitable step size, μ_opt_, both the Armijo^41,44^ and Wolfe line search^43^ conditions were implemented. If the line search along the l-BFGS direction fails, the search direction is reset to the SA vector, and the line search is then repeated.

The unitary matrices at each **k** point are updated,
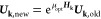
29until the norm of the Riemannian gradient decreases below a threshold. The output unitary can then be applied directly to the original Bloch functions to obtain localized WFs on the *B*_1_ basis using eq 8.

## 3 Results

### 3.1 Computational Details

The Bloch IAO procedure and the PMWF localization have been implemented in a developmental version of the TURBOMOLE^25^ package. The initial mean-field Bloch functions were obtained through the periodic HF procedure within the riper module.^45−49^ To generate the IAOs, a minimal basis was constructed from HF calculations of isolated atoms in the cc-pVTZ^50,51^ basis, as already implemented within TURBOMOLE to construct molecular IAOs. The PM functional was evaluated with a penalty exponent of *p* = 4, rather than 2, as shown in eq 10, due to better localization for π character orbitals, as discussed in prior works.^26,27^

An Armijo step size method and a Wolfe line search were tested in the localization procedure. It is known that a line search fulfilling the Wolfe conditions ensures stability of the BFGS updates, by ensuring the approximate Hessian, within our maximization problem, is negative definite.^43^ However, computation of the line search is costly since multiple gradient evaluations are required along the trial direction. By contrast, the Armijo line search only requires PM metric values to be evaluated, and we observed its convergence performance to be similar to that of the Wolfe line search, with shorter wall times. The Armijo search was employed in all calculations subsequently discussed in this article.

Table 1 details the insulating and semiconducting systems used to probe the performance of the IAO PM localization scheme. The original basis set, *B*_1_, used in the mean-field calculation, and the Monkhorst–Pack^35^ mesh, are shown. For the RI-J approximation, all test systems utilized the universal Coulomb-fitting auxiliary basis sets^53^ with the exception of the magnesium oxide and carbon nanotube systems, which employed auxiliary functions optimized for the def-SVP and def2-SVP basis sets, respectively.^53^ Unit cell parameters and geometries are provided in the Supporting Information. All figures were plotted using Avogadro.^54^

**Table 1:** Insulating and Semiconducting Systems used To Test the Bloch IAO and PM Localization Procedure

system	basis *B*_1_	Monkhorst–Pack mesh size
diamond	pob-TZVP^52^	11,11,11
silicon	pob-TZVP	11,11,11
boron nitride	pob-TZVP	15,15
graphene	pob-TZVP	15,15
MgO	def-SVP	11,11,11
SiO_2_	pob-TZVP	5,5,5
*trans*-(C_2_H_2_)_*∞*_	pob-TZVP	101
(4,4) C-nanotube	def2-SVP	11

### 3.2 Overall Performance

Table 2 reports the performance of the Bloch IAO localization scheme. The values of the PM metric are presented for the WFs of the SCF calculation, after rotation into the natural gauge, after diabatic Wannierization, and after PM optimization. The number of iterations required to localize the PM objective function with l-BFGS, compared to SA, using diabatic WFs as the initial guess, is also given. The convergence threshold for the PM gradient norm was set to 1 × 10^–5^, with the exception of the threshold for the boron nitride (BN) system, which was set to 1 × 10^–6^. All occupied orbitals are included in the optimization.

**Table 2:** PM Metric Values of WFs from the Initial SCF Calculation, after Rotation into the Natural Gauge, after Diabatic Wannierization, and after PM Optimization^a^

system	⟨O⟩_PM_ (SCF)	⟨O⟩_PM_ (Nat.)	⟨O⟩_PM_ (Dia.)	⟨O⟩_PM_ (Opt.)	l-BFGS	SA
diamond	1.49 × 10^–8^	6.13 × 10^–2^	1.92	2.66	35	100
silicon	2.40 × 10^–2^	6.64 × 10^–2^	9.79	10.66	42	114
boron nitride	1.20 × 10^–5^	2.36	3.19	3.37	55	731
graphene	3.37 × 10^–6^	8.37 × 10^–2^	1.94	2.56	42	60
MgO	2.88 × 10^–1^	3.87	9.56	9.61	8	744
SiO_2_	5.96 × 10^–4^	9.55	30.80	37.29	338	1794
*trans*-(C_2_H_2_)_*∞*_	4.92 × 10^–5^	4.25 × 10^–2^	2.17	2.76	43	1517
(4,4) C-nanotube	1.65 × 10^–3^	4.34 × 10^–3^	33.70	40.60	62	261

a The number of iterations to converge PM metric, after the initial diabatic preparation, using l-BFGS or SA.

In all cases, the final values of the PM metric from the l-BFGS and SA optimizations were equal, to a precision of 10^–5^ (or 10^–6^ for BN), confirming that the output WFs were equally localized. A tighter threshold was required for BN in order for the output WFs from l-BFGS and SA to agree to target precision. As demonstrated by Clement et al.,^15^ we confirm that utilizing l-BFGS, compared to SA, markedly improves convergence performance. In some systems, a 10-fold reduction in iterations to converge is observed, if not greater. The PM optimizer performs robustly across the range of insulating and semiconducting materials explored, successfully localizing every test system. With the exception of silicon dioxide, all the test systems converge within 100 iterations with l-BFGS. A larger number of iterations were required for silicon dioxide. We observed that the step size was very small, which indicates that the Hessian description of the landscape in this case may be poor. Despite this, l-BFGS still converges five times faster compared to SA, showing the robustness of the scheme.

We stress that using diabatic WFs as the initial preparation has an important role in the robustness and quality of the final localized WFs. As seen in Table 2, the values of the PM metric after diabatic Wannierization are remarkably close to the final optimized values, showing that a significant degree of locality has been captured through the diabatization. Our experiments using randomly generated unitary matrices as the initial guess led to final WFs with metric values that consistently were smaller than that obtained from the diabatic preparation and never greater. The choice of objective functional, and the parametrization employed for the gradient, gives an optimization landscape with many local maxima, and the use of an appropriate initial guess, such as diabatic WFs, is required to ensure that higher valued maxima are located, compared to random unitary sampling. Direct comparison of the number of iterations required to converge, using a random guess and the diabatic preparation, is often not possible since the final WFs are usually inequivalent. Although we have not verified it in this work, we predict that localizing the Bloch functions of the Γ-point with an IAO procedure instead of via the Cholesky decomposition would further increase ⟨*O*⟩_**PM**_(Dia.) and would reduce the number of iterations required for full optimization.

The initial values of the PM metric from direct Wannierization of the SCF Bloch functions are very small. It should be noted that due to the different gauges of the Bloch functions in separate calculations, the values for ⟨O⟩_**PM**_(SCF) can vary arbitrarily. The values reported in Table 2 are, in fact, the largest value of ⟨O⟩_**PM**_(SCF) taken from 10 separate calculations. Throughout our testing, we have never observed any example where ⟨O⟩_**PM**_(SCF) has been greater or similar in magnitude to ⟨O⟩_**PM**_(Dia.). Applying the natural gauge to the Bloch functions increases the values of the PM metric in all cases, confirming that the natural gauge smooths the Bloch functions in the reciprocal space. For crystals with well-separated bands formed from weakly interacting AOs, simply applying the natural gauge results in well-localized WFs. In all cases, ⟨O⟩_**PM**_(Nat.) sits in between the SCF and diabatic values.

**Figure 1:**
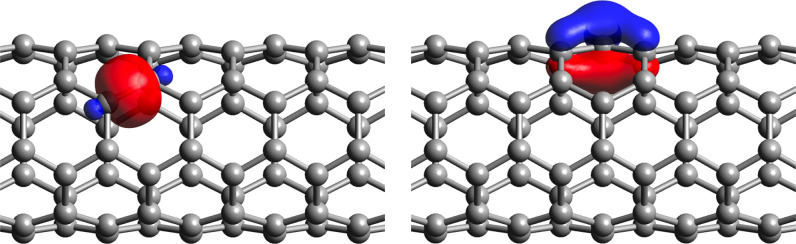
Bloch IAO localized WFs of the (4,4) nanotube, showing σ (left) and π (right) bonding characters. An isosurface value of 0.05 was used.

Figure 1 presents an example of localized WFs of the (4,4) nanotube system, described in Table 1. The left subfigure clearly shows a carbon–carbon σ bond, while the right subfigure illustrates a π character WF. This π and σ separation is observed in the other test systems, demonstrating that the Bloch IAO localization procedure retains the advantages of the original PM metric.

### 3.3 Separate Optimization of Valence and Core Bands

It is often desirable to localize WFs for the core and valence bands separately. For example, the accurate calculation of correlation energies within local correlation methods requires localized occupied orbitals without contributions from uncorrelated core orbitals.^55^ In view of this, the Bloch IAO localization scheme performs exceptionally well in localizing core and valence bands independently. This is primarily due to the quality of the diabatic WFs, which almost immediately generate localized core orbitals.

Table 3 shows the performance of the Bloch IAO localization, with core and valence band separation, for all test systems. The gradient norm of the PM metric for the core bands after the diabatic initial preparation is presented. Subsequent l-BFGS iterations required to localize using the PM metric for both the core and valence bands are also given. The PM gradient norms of our initial core WFs are all already close to the convergence threshold (10^–5^, or 10^–6^ for BN) and localize within 3 l-BFGS iterations, demonstrating that the diabatic preparation yields nearly optimally local core WFs. The number of iterations required to localize the core increases typically only by 1 or 2 iterations when the convergence threshold is increased to 10^–8^. Across all test systems, the difference in the value of the PM metric between the final localized core orbitals and *n*_core_ was within 3 × 10^–2^, where *n*_core_ is given by eq 12, summing only over core bands. This indicates that the core orbitals are localized to the global maxima, given that *n*_core_ is the upper bound for the PM metric for the core. Localization of the valence bands also occurs rapidly, although less markedly than in the core case, and the total number of iterations required to localize the core and valence bands separately is less than that required to localize the full occupied space (Table 2), across all the test systems. This is to be expected, given that the dimensionality of the optimization problem is reduced by separating the core and valence bands.

**Table 3:** Number of Iterations To Converge PM Metric with an Initial Diabatic Preparation, with Core and Valence Band Separation

system	initial core PM gradient	core	valence
diamond	1.08 × 10^–4^	2	20
silicon	8.73 × 10^–4^	3	24
boron nitride	9.70 × 10^–4^	3	27
graphene	1.91 × 10^–4^	2	22
MgO	5.17 × 10^–5^	2	4
SiO2	6.55 × 10^–3^	2	113
*trans*-(C_2_H_2_)_*∞*_	1.67 × 10^–4^	3	22
(4,4) C-nanotube	6.81 × 10^–2^	3	29

Diabatic preparation is particularly effective in localizing WFs from bands composed of weakly interacting AOs. The core bands and valence bands with a strong ionic character, for example, in MgO, require only a few optimization steps for optimization. Since the localized valence WFs are usually bonding in character, they are typically centered between atoms and inherently less local than their core counterparts and require more steps for optimization. Even for the core bands, using diabatic WFs as an initial preparation is vital for the robustness and accuracy of the Bloch IAO scheme. Using a random initial unitary for core bands frequently leads to output WFs with PM metric values significantly smaller than those obtained with the diabatic guess, showing that encountering local maxima is a common problem without the correct preparation of the WFs.

### 3.4 Chemical Intuition of Bloch IAO-Generated WFs

One of the key strengths of the original IAO scheme is the clear interpretation of these MOs and the direct connection to chemical intuition and concepts. Knizia^26^ demonstrated that IAOs allow robust, basis set independent, partial charges, and orbital populations to be computed. This enables quantitative measures for electronegativities and oxidation states for molecules, which align with empirical understanding, to be evaluated. In the analogous fashion, we demonstrate that Bloch IAO-localized WFs provide chemical understanding in periodic systems. Bloch IAO partial charges can be computed in a similar fashion to molecular IAOs, by summing the atomic contributions across all the cells within the supercell,
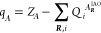
30where *Z*_*A*_ is the atom’s nuclear charge. IAO partial charges can be computed for any periodic system such as those listed in Table 1. However, it is also worth stressing that IAOs can be used for both periodic and molecular systems, on equal footing, meaning that they are a robust and consistent partial charge estimate for probing both material and molecular systems. This opens the possibility to investigate interesting chemical scenarios, such as systems containing interactions between materials and molecules.

The adsorption of CO onto the MgO(001) surface has been heralded as the ‘hydrogen molecule of surface science’,^56^ and an important case study for the theoretical understanding of heterogeneous catalysis. Obtaining accurate adsorption energy for this system remains a highly discussed topic, in which many quantum-chemical and many-body methods have been utilized,^57−61^ in order to achieve consensus with experimental data. This adsorption example is an ideal case to demonstrate the ability that Bloch IAO-localized WFs have to provide insights into the underlying chemistry of the system.

To model the system, a unit cell consisting of a 4 × 4 × 1 slab of MgO was constructed. CO, orientated perpendicular to the surface, was positioned with a C–Mg equilibrium distance of 2.479 Å.^58^ To obtain mean-field Bloch functions, a periodic DFT (PBE^62^) calculation was conducted in the riper module, using the pob-TZVP^52^ basis set, on a (3,3) Monkhorst–Pack mesh to sample the Brillouin zone. Bloch IAO-localized WFs were then obtained.

Although higher-level quantum chemical methods have been used elsewhere to attempt to accurately model the weak van der Waals interactions dominating the adsorption, we stress that our motivation is to use this system to exhibit the use of IAO WFs for chemical intuition. Since orbitals provide a zeroth-order description for the motion of the electrons and are a result from mean-field, effective one-electron theories, using solely DFT to model this picture serves our purposes.

**Figure 2:**
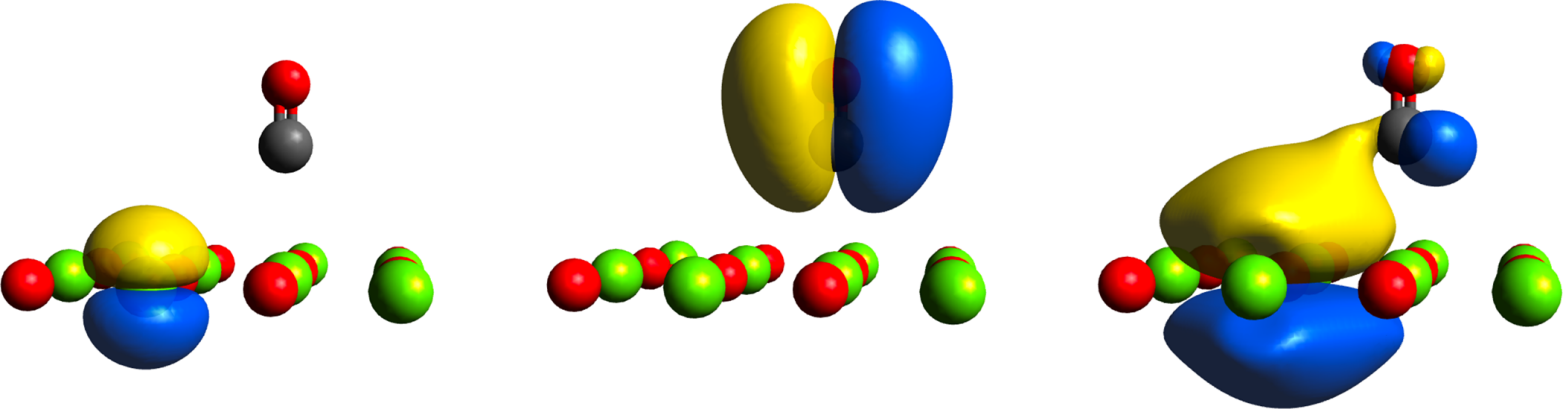
Bloch IAO-localized WFs of the MgO(001) CO adsorption system. A magnesium 2p-like orbital is shown (left), as well as a π bonding orbital on CO (center). A WF demonstrating oxygen 3p orbital donation, on the MgO surface, to the CO π* orbital is also presented (right). Only atoms in the reference cell are shown. An isosurface value of 0.01 was used.

Figure 2 presents three Bloch IAO-localized WFs of the system, all three of which align with chemical understanding. The left and center subfigures show a localized 2p orbital, centered on Mg, and a π bonding orbital, centered on CO, respectively. Similar WFs are observed when WFs for the surface and adsorbate are computed separately. A more interesting WF is presented on the right, where back-bonding from the nearest neighbor oxygen atom, on the MgO surface, to the π* orbital of carbon monoxide, is shown. Although the role of back-bonding within metal oxide adsorption has been scrutinized,^61,63^ we wish to make the point that IAO-localized WFs provide direct and intuitive chemical understanding, consistent with the level of theory used to generate the original Bloch functions.

The Bloch IAO partial charges of the MgO(001) CO adsorption system, in the equilibrium geometry, were also calculated. In Table 4, we compare these partial charge values to charges obtained from separate periodic calculations of the MgO surface slab and the CO molecule, representing a noninteracting scenario. Most significantly, a reduction in the positive partial charge on the carbon of CO is observed moving from the noninteracting to equilibrium geometry, as well as a decrease in the negative charge on the oxygen nearest neighbors of MgO. This can be rationalized from the back-bonding process presented in Figure 2, which once again shows that quantitative measures, such as Bloch IAO partial charges, are consistent with chemical intuition at the level of theory employed in the mean-field picture.

**Table 4:** Bloch IAO Partial Charges of the Noninteracting and Equilibrium MgO(001) CO Adsorption System

system	C (CO)	O (CO)	Mg (MgO)	O (MgO)
noninteracting	0.42	–0.42	1.68	–1.68
equilibrium	0.32	–0.41	1.70^a^	–1.65^b^

a The partial charge of the closest Mg atom to CO is given.

b The average partial charge of the four nearest neighbors to CO is given.

## 4 Conclusions

We have generalized the IAO method to periodic solids. Bloch IAOs form a minimal basis that exactly represents the occupied bands and thus removes the well-known issue of using Mulliken charges for nonminimal basis sets. They thus enable localized WFs, optimized using the PM metric, to be robustly evaluated, as first introduced by Jónsson et al.^14^ We outline a localization scheme, which prepares the initial Bloch functions by diabatically transferring locality imposed at the gamma point through the Brillouin zone, before localizing according to the PM metric. Clement et al.^15^ demonstrated improved performance using l-BFGS compared to other gradient-based solvers, and we confirm this. This scheme works efficiently across a range of semiconducting and insulating solids, and in particular, we highlight the ability of the diabatic WFs to localize atom-centered WFs almost immediately. Using the example of CO adsorption onto MgO(001), we demonstrate that Bloch IAO partial charges can provide chemical insights into systems, through visualization of the localized WFs and through computing measures such as partial charges. We expect that Bloch IAOs will provide a bridge for understanding chemical phenomena within periodic systems. Bloch IAOs are not solely restricted to LCAO methods, but can also be applied with plane-wave basis sets by computing the overlap between plane-waves and the minimal Gaussian AO basis.^32^ In particular, we note that in the molecular setting, localized MOs using IAOs have proved popular in constructing localized occupied orbitals and domains for use within local correlation theories.^55^ Bloch IAOs may provide an analogous route for similar implementations within periodic systems.

## Supporting Information Available

Unit cell parameters and geometries for all test systems (PDF)

## References

[ref1] Hohenberg P.; Kohn W. Inhomogeneous electron gas. Phys. Rev. 1964, 136, B864 10.1103/PhysRev.136.B864.

[ref2] Kohn W.; Sham L. J. Self-consistent equations including exchange and correlation effects. Phys. Rev. 1965, 140, A1133 10.1103/PhysRev.140.A1133.

[ref3] Wannier G. H. The structure of electronic excitation levels in insulating crystals. Phys. Rev. 1937, 52, 191 10.1103/PhysRev.52.191.

[ref4] Saebo S.; Pulay P. Local treatment of electron correlation. Annu. Rev. Phys. Chem. 1993, 44, 213 10.1146/annurev.pc.44.100193.001241.

[ref5] Hampel C.; Werner H. Local treatment of electron correlation in coupled cluster theory. J. Chem. Phys. 1996, 104, 6286 10.1063/1.471289.

[ref6] Maschio L.; Usvyat D.; Manby F. R.; Casassa S.; Pisani C.; Schütz M. Fast local-mp2 method with density-fitting for crystals. i. theory and algorithms. Phys. Rev. B 2007, 76, 075101 10.1103/PhysRevB.76.075101.

[ref7] Usvyat D.; Civalleri B.; Maschio L.; Dovesi R.; Pisani C.; Schütz M. Approaching the theoretical limit in periodic local MP2 calculations with atomic-orbital basis sets: The case of LiH. J. Chem. Phys. 2011, 134, 214105 10.1063/1.3595514. 21663342

[ref8] Boys S. F. Construction of some molecular orbitals to be approximately invariant for changes from one molecule to another. Rev. Mod. Phys. 1960, 32, 296 10.1103/RevModPhys.32.296.

[ref9] Foster J. M.; Boys S. F. Canonical configurational interaction procedure. Rev. Mod. Phys. 1960, 32, 300 10.1103/RevModPhys.32.300.

[ref10] Pipek J.; Mezey P. G. A fast intrinsic localization procedure applicable for ab initio and semiempirical linear combination of atomic orbital wave functions. J. Chem. Phys. 1989, 90, 4916 10.1063/1.456588.

[ref11] Marzari N.; Vanderbilt D. Maximally localized generalized wannier functions for composite energy bands. Phys. Rev. B 1997, 56, 12847 10.1103/PhysRevB.56.12847.

[ref12] Pizzi G.; Vitale V.; Arita R.; Blügel S.; Freimuth F.; Géranton G.; Gibertini M.; Gresch D.; Johnson C.; Koretsune T.; Ibañez-Azpiroz J.; Lee H.; Lihm J.-M.; Marchand D.; Marrazzo A.; Mokrousov Y.; Mustafa J. I.; Nohara Y.; Nomura Y.; Paulatto L.; Poncé S.; Ponweiser T.; Qiao J.; Thöle F.; Tsirkin S. S.; Wierzbowska M.; Marzari N.; Vanderbilt D.; Souza I.; Mostofi A. A.; Yates J. R. Wannier90 as a community code: new features and applications. J. Phys.: Condens. Matter 2020, 32, 165902 10.1088/1361-648X/ab51ff. 31658458

[ref13] Marzari N.; Mostofi A. A.; Yates J. R.; Souza I.; Vanderbilt D. Maximally localized wannier functions: Theory and applications. Rev. Mod. Phys. 2012, 84, 1419 10.1103/RevModPhys.84.1419.

[ref14] Jónsson E. O.; Lehtola S.; Puska M.; Jónsson H. Theory and applications of generalized pipek–mezey wannier functions. J. Chem. Theory Comput. 2017, 13, 460 10.1021/acs.jctc.6b00809. 28099002

[ref15] Clement M. C.; Wang X.; Valeev E. F. Robust pipek–mezey orbital localization in periodic solids. J. Chem. Theory Comput. 2021, 17, 7406 10.1021/acs.jctc.1c00238. 34739235

[ref16] Schreder L.; Luber S. Propagated (fragment) Pipek–Mezey Wannier functions in real-time time-dependent density functional theory. J. Chem. Phys. 2024, 160, 214117 10.1063/5.0203442. 38832736

[ref17] Kresse G.; Hafner J. Ab initio molecular dynamics for liquid metals. Phys. Rev. B 1993, 47, 558 10.1103/PhysRevB.47.558. 10004490

[ref18] Giannozzi P.; Andreussi O.; Brumme T.; Bunau O.; Nardelli M. B.; Calandra M.; Car R.; Cavazzoni C.; Ceresoli D.; Cococcioni M.; Colonna N.; Carnimeo I.; Corso A. D.; de Gironcoli S.; Delugas P.; R A. D. Jr; Ferretti A.; Floris A.; Fratesi G.; Fugallo G.; Gebauer R.; Gerstmann U.; Giustino F.; Gorni T.; Jia J.; Kawamura M.; Ko H.-Y.; Kokalj A.; Küçükbenli E.; Lazzeri M.; Marsili M.; Marzari N.; Mauri F.; Nguyen N. L.; Nguyen H.-V.; de-la Roza A. O.; Paulatto L.; Poncé S.; Rocca D.; Sabatini R.; Santra B.; Schlipf M.; Seitsonen A. P.; Smogunov A.; Timrov I.; Thonhauser T.; Umari P.; Vast N.; Wu X.; Baroni S. Advanced capabilities for materials modelling with quantum espresso. J. Phys.: Condens. Matter 2017, 29, 465901 10.1088/1361-648X/aa8f79. 29064822

[ref19] Clark S. J.; Segall M. D.; Pickard C. J.; Hasnip P. J.; Probert M. I. J.; Refson K.; Payne M. C. First principles methods using castep. Zeitschrift für Kristallographie - Crystalline Materials 2005, 220, 567 10.1524/zkri.220.5.567.65075.

[ref20] Gonze X.; Jollet F.; Abreu Araujo F.; Adams D.; Amadon B.; Applencourt T.; Audouze C.; Beuken J.-M.; Bieder J.; Bokhanchuk A.; Bousquet E.; Bruneval F.; Caliste D.; Côté M.; Dahm F.; Da Pieve F.; Delaveau M.; Di Gennaro M.; Dorado B.; Espejo C.; Geneste G.; Genovese L.; Gerossier A.; Giantomassi M.; Gillet Y.; Hamann D.; He L.; Jomard G.; Laflamme Janssen J.; Le Roux S.; Levitt A.; Lherbier A.; Liu F.; Lukačević I.; Martin A.; Martins C.; Oliveira M.; Poncé S.; Pouillon Y.; Rangel T.; Rignanese G.-M.; Romero A.; Rousseau B.; Rubel O.; Shukri A.; Stankovski M.; Torrent M.; Van Setten M.; Van Troeye B.; Verstraete M.; Waroquiers D.; Wiktor J.; Xu B.; Zhou A.; Zwanziger J. Recent developments in the abinit software package. Comput. Phys. Commun. 2016, 205, 106 10.1016/j.cpc.2016.04.003.

[ref21] Erba A.; Desmarais J. K.; Casassa S.; Civalleri B.; Donà L.; Bush I. J.; Searle B.; Maschio L.; Edith-Daga L.; Cossard A.; Ribaldone C.; Ascrizzi E.; Marana N. L.; Flament J.-P.; Kirtman B. Crystal23: A program for computational solid state physics and chemistry. J. Chem. Theory Comput. 2023, 19, 6891 10.1021/acs.jctc.2c00958. 36502394 PMC10601489

[ref22] Pisani C.; Dovesi R. Exact-exchange hartree–fock calculations for periodic systems. i. illustration of the method. Int. J. Quantum Chem. 1980, 17, 501 10.1002/qua.560170311.

[ref23] Dovesi R.; Pisani C.; Roetti C. Exact-exchange hartree–fock calculations for periodic systems. ii. results for graphite and hexagonal boron nitride. Int. J. Quantum Chem. 1980, 17, 517.

[ref24] Sun Q.; Zhang X.; Banerjee S.; Bao P.; Barbry M.; Blunt N. S.; Bogdanov N. A.; Booth G. H.; Chen J.; Cui Z.-H.; et al. Recent developments in the pyscf program package. J. Chem. Phys. 2020, 153, 024109 10.1063/5.0006074. 32668948

[ref25] Balasubramani S. G.; Chen G. P.; Coriani S.; Diedenhofen M.; Frank M. S.; Franzke Y. J.; Furche F.; Grotjahn R.; Harding M. E.; Hättig C.; Hellweg A.; Helmich-Paris B.; Holzer C.; Huniar U.; Kaupp M.; Marefat Khah A.; Karbalaei Khani S.; Müller T.; Mack F.; Nguyen B. D.; Parker S. M.; Perlt E.; Rappoport D.; Reiter K.; Roy S.; Rückert M.; Schmitz G.; Sierka M.; Tapavicza E.; Tew D. P.; van Wüllen C.; Voora V. K.; Weigend F.; Wodyński A.; Yu J. M. Turbomole: Modular program suite for ab initio quantum-chemical and condensed-matter simulations. J. Chem. Phys. 2020, 152, 184107 10.1063/5.0004635. 32414256 PMC7228783

[ref26] Knizia G. Intrinsic atomic orbitals: An unbiased bridge between quantum theory and chemical concepts. J. Chem. Theory Comput. 2013, 9, 4834 10.1021/ct400687b. 26583402

[ref27] Lehtola S.; Jónsson H. Pipek–mezey orbital localization using various partial charge estimates. J. Chem. Theory Comput. 2014, 10, 642 10.1021/ct401016x. 26580041

[ref28] Cioslowski J. Partitioning of the orbital overlap matrix and the localization criteria. J. Math. Chem. 1991, 8, 169 10.1007/BF01166933.

[ref29] Alcoba D. R.; Lain L.; Torre A.; Bochicchio R. C. An orbital localization criterion based on the theory of “fuzzy” atoms. J. Comput. Chem. 2006, 27, 596 10.1002/jcc.20373. 16470667

[ref30] Werner H.-J.; Knowles P. J.; Manby F. R.; Black J. A.; Doll K.; Heßelmann A.; Kats D.; Köhn A.; Korona T.; Kreplin D. A.; et al. The molpro quantum chemistry package. J. Chem. Phys. 2020, 152, 144107 10.1063/5.0005081. 32295355

[ref31] Saue T.; Bast R.; Gomes A. S. P.; Jensen H. J. A.; Visscher L.; Aucar I. A.; Di Remigio R.; Dyall K. G.; Eliav E.; Fasshauer E.; et al. The dirac code for relativistic molecular calculations. J. Chem. Phys. 2020, 152, 204104 10.1063/5.0004844. 32486677

[ref32] Schäfer T.; Gallo A.; Irmler A.; Hummel F.; Grüneis A. Surface science using coupled cluster theory via local Wannier functions and in-RPA-embedding: The case of water on graphitic carbon nitride. J. Chem. Phys. 2021, 155, 244103 10.1063/5.0074936. 34972356

[ref33] Janowski T. Near equivalence of intrinsic atomic orbitals and quasiatomic orbitals. J. Chem. Theory Comput. 2014, 10, 3085 10.1021/ct500245f. 26588279

[ref34] Cui Z.-H.; Zhu T.; Chan G. K.-L. Efficient implementation of ab initio quantum embedding in periodic systems: Density matrix embedding theory. J. Chem. Theory Comput. 2020, 16, 119 10.1021/acs.jctc.9b00933. 31815466

[ref35] Monkhorst H. J.; Pack J. D. Special points for brillouin-zone integrations. Phys. Rev. B 1976, 13, 5188 10.1103/PhysRevB.13.5188.

[ref36] Lehtola S.; Jónsson H. Unitary optimization of localized molecular orbitals. J. Chem. Theory Comput. 2013, 9, 5365 10.1021/ct400793q. 26592274

[ref37] Cardoso J.-F.; Souloumiac A. Jacobi angles for simultaneous diagonalization. SIAM Journal on Matrix Analysis and Applications 1996, 17, 161 10.1137/S0895479893259546.

[ref38] Horn R. A.; Johnson C. R. Matrix analysis; Cambridge university press, 2012.

[ref39] Gower J. C.; Dijksterhuis G. B. Procrustes problems; OUP Oxford, 2004; Vol. 30.

[ref40] Abrudan T.; Eriksson J.; Koivunen V. Conjugate gradient algorithm for optimization under unitary matrix constraint. Signal Processing 2009, 89, 1704 10.1016/j.sigpro.2009.03.015.

[ref41] Abrudan T. E.; Eriksson J.; Koivunen V. Steepest descent algorithms for optimization under unitary matrix constraint. IEEE Transactions on Signal Processing 2008, 56, 1134 10.1109/TSP.2007.908999.

[ref42] Hyvärinen A.; Karhunen J.; Oja E. Independent Component Analysis, Adaptive and Cognitive Dynamic Systems: Signal Processing, Learning, Communications and Control; Wiley, 2001.

[ref43] Nocedal J.; Wright S. J. Numerical Optimization, 2nd ed.; Springer: New York, NY, USA, 2006.

[ref44] Polak E. Optimization: Algorithms and Consistent Approximations; Springer-Verlag: Berlin, Heidelberg, 1997.

[ref45] Łazarski R.; Burow A. M.; Sierka M. Density functional theory for molecular and periodic systems using density fitting and continuous fast multipole methods. J. Chem. Theory Comput. 2015, 11, 3029 10.1021/acs.jctc.5b00252. 26575740

[ref46] Łazarski R.; Burow A. M.; Grajciar L.; Sierka M. Density functional theory for molecular and periodic systems using density fitting and continuous fast multipole method: Analytical gradients. J. Comput. Chem. 2016, 37, 2518 10.1002/jcc.24477. 27555218

[ref47] Burow A. M.; Sierka M. Linear scaling hierarchical integration scheme for the exchange-correlation term in molecular and periodic systems. J. Chem. Theory Comput. 2011, 7, 3097 10.1021/ct200412r. 26598153

[ref48] Burow A. M.; Sierka M.; Mohamed F. Resolution of identity approximation for the Coulomb term in molecular and periodic systems. J. Chem. Phys. 2009, 131, 214101 10.1063/1.3267858. 19968331

[ref49] Müller C.; Sharma M.; Sierka M. Real-time time-dependent density functional theory using density fitting and the continuous fast multipole method. J. Comput. Chem. 2020, 41, 2573 10.1002/jcc.26412. 33464600

[ref50] Dunning T. Gaussian basis sets for use in correlated molecular calculations. i. the atoms boron through neon and hydrogen. J. Chem. Phys. 1989, 90, 1007 10.1063/1.456153.

[ref51] Prascher B. P.; Woon D. E.; Peterson K. A.; Dunning T. H.; Wilson A. K. Gaussian basis sets for use in correlated molecular calculations. vii. valence, core-valence, and scalar relativistic basis sets for li, be, na, and mg. Theor. Chem. Acc. 2011, 128, 69 10.1007/s00214-010-0764-0.

[ref52] Peintinger M. F.; Oliveira D. V.; Bredow T. Consistent gaussian basis sets of triple-zeta valence with polarization quality for solid-state calculations. J. Comput. Chem. 2013, 34, 451 10.1002/jcc.23153. 23115105

[ref53] Weigend F. Accurate coulomb-fitting basis sets for h to rn. Phys. Chem. Chem. Phys. 2006, 8, 1057 10.1039/b515623h. 16633586

[ref54] Hanwell M. D.; Curtis D. E.; Lonie D. C.; Vandermeersch T.; Zurek E.; Hutchison G. R. Avogadro: an advanced semantic chemical editor, visualization, and analysis platform. J. Cheminf. 2012, 4, 17 10.1186/1758-2946-4-17. PMC3542060 22889332

[ref55] Ma Q.; Werner H.-J. Explicitly correlated local coupled-cluster methods using pair natural orbitals. WIREs Comput. Mol. Sci. 2018, 8, e1371 10.1002/wcms.1371. 29211961

[ref56] Sauer J. Ab initio calculations for molecule–surface interactions with chemical accuracy. Acc. Chem. Res. 2019, 52, 3502 10.1021/acs.accounts.9b00506. 31765121

[ref57] Nygren M. A.; Pettersson L. G. M. Comparing ab initio computed energetics with thermal experiments in surface science: CO/MgO(001). J. Chem. Phys. 1996, 105, 9339 10.1063/1.472724.

[ref58] Mitra A.; Hermes M. R.; Cho M.; Agarawal V.; Gagliardi L. Periodic density matrix embedding for co adsorption on the mgo(001) surface. J. Phys. Chem. Lett. 2022, 13, 7483 10.1021/acs.jpclett.2c01915. 35939641 PMC9393885

[ref59] Shi B. X.; Zen A.; Kapil V.; Nagy P. R.; Grüneis A.; Michaelides A. Many-body methods for surface chemistry come of age: Achieving consensus with experiments. J. Am. Chem. Soc. 2023, 145, 25372 10.1021/jacs.3c09616. 37948071 PMC10683001

[ref60] Ye H.-Z.; Berkelbach T. C. Co adsorption on the surface of mgo from periodic coupled-cluster theory with local natural orbitals: Adding to the consensus (2023), arXiv:2309.14651 [cond-mat.mtrl-sci].

[ref61] Neyman K. M.; Rösch N. Co bonding and vibrational modes on a perfect mgo(001) surface: Lcgto-ldf model cluster investigation. Chem. Phys. 1992, 168, 267 10.1016/0301-0104(92)87161-2.

[ref62] Perdew J. P.; Burke K.; Ernzerhof M. Generalized gradient approximation made simple. Phys. Rev. Lett. 1996, 77, 3865 10.1103/PhysRevLett.77.3865. 10062328

[ref63] Pacchionia G.; Neyman K. M.; Rosch N. Co adsorption on the (001) surface of mgo: a comparison of hartree-fock and local density functional results. J. Electron Spectrosc. Relat. Phenom. 1994, 69, 13 10.1016/S0368-2048(14)80004-2.

